# The Elusive Anti-*Candida* Vaccine: Lessons From the Past and Opportunities for the Future

**DOI:** 10.3389/fimmu.2018.00897

**Published:** 2018-04-27

**Authors:** Gloria Hoi Wan Tso, Jose Antonio Reales-Calderon, Norman Pavelka

**Affiliations:** Singapore Immunology Network (SIgN), Agency of Science, Technology and Research (A*STAR), Singapore, Singapore

**Keywords:** *Candida*, candidemia, candidiasis, vaccine, trained immunity, opportunistic infections, immunocompromised patients

## Abstract

Candidemia is a bloodstream fungal infection caused by *Candida* species and is most commonly observed in hospitalized patients. Even with proper antifungal drug treatment, mortality rates remain high at 40–50%. Therefore, prophylactic or preemptive antifungal medications are currently recommended in order to prevent infections in high-risk patients. Moreover, the majority of women experience at least one episode of vulvovaginal candidiasis (VVC) throughout their lifetime and many of them suffer from recurrent VVC (RVVC) with frequent relapses for the rest of their lives. While there currently exists no definitive cure, the only available treatment for RVVC is again represented by antifungal drug therapy. However, due to the limited number of existing antifungal drugs, their associated side effects and the increasing occurrence of drug resistance, other approaches are greatly needed. An obvious prevention measure for candidemia or RVVC relapse would be to immunize at-risk patients with a vaccine effective against *Candida* infections. In spite of the advanced and proven techniques successfully applied to the development of antibacterial or antiviral vaccines, however, no antifungal vaccine is still available on the market. In this review, we first summarize various efforts to date in the development of anti-*Candida* vaccines, highlighting advantages and disadvantages of each strategy. We next unfold and discuss general hurdles encountered along these efforts, such as the existence of large genomic variation and phenotypic plasticity across *Candida* strains and species, and the difficulty in mounting protective immune responses in immunocompromised or immunosuppressed patients. Lastly, we review the concept of “trained immunity” and discuss how induction of this rapid and nonspecific immune response may potentially open new and alternative preventive strategies against opportunistic infections by *Candida* species and potentially other pathogens.

## Introduction

Due to advances in medicine and surgery, over the past century there has been a rising population of immunocompromised patients, which are at an elevated risk to suffer from opportunistic infections (1,  2). While hospital-acquired fungal infections are less frequent than bacterial ones, they disproportionately account for higher mortality rates, longer hospitalization times and increased healthcare costs (3). Risk factors for fungal infections are very broad and nonspecific, and they include chronic respiratory disease, cancer, HIV infection, organ transplantation, neutropenia, presence of central venous catheters, prolonged hospital stay, administration of total parenteral nutrition, exposure to invasive procedures, chemotherapy, hemodialysis, gastric acid suppression, and use of broad-spectrum antibiotics (4, 5). Candidiasis (caused by *Candida* species) is the most common opportunistic fungal infection and the fourth most common nosocomial bloodstream infection (6, 7). Despite the best available standards of care, the incidence of candidemia, a sign of invasive or systemic candidiasis, is on the rise in the US (8) and mortality rates often exceed 50% despite use of antifungal drugs (9). This is especially true in intensive care units and in immunocompromised patients, where *Candida* bloodstream infections are estimated to strike ~400,000 patients a year, with an associated mortality of 46–75% (10). For all these reasons, and because the hospitalization and treatment cost of these patients is very high (3, 11), CDC guidelines recommend prophylactic or preemptive antifungal treatment in patients considered at high risk of candidemia (12).

Mucosal fungal infections, though almost never life-threatening, are much more common than invasive ones and can be associated with high morbidity, socioeconomic impact, and low quality of life. The most common infection sites are the oral cavity and the genitourinary tract (13). Vulvovaginal candidiasis (VVC) is the most common mucosal fungal infection, with different studies estimating that 50–70% of women suffer an episode of VVC at least once in their lifetime and 5–8% of women suffer from recurrent VVC (RVVC) (14). To this date, there still exists no definitive cure for RVVC, which is commonly treated with over-the-counter antifungal medications but continues to relapse ≥4 times a year for the rest of a woman’s life (15).

Antifungal agents available for the treatment of systemic or mucosal candidiasis are restricted to only a few classes of compounds, such as azoles, polyenes, allylamines, and echinocandins (16). However, adverse side effects, toxicity, and emergence of drug resistance limit the use of these drugs (10). In particular, various *Candida* species can acquire resistance to different antifungals or, even worse, to more than one drug (17–20). In fact, the emergence of drug resistance in *Candida* spp. has been on a growing trend over the past decade (21), with multidrug-resistant *Candida* spp. now being reported all over the world (22, 23). Finally, the recent discovery of a novel multidrug resistant species of *Candida* (*C. auris*) poses a further threat to our ability to use antifungal drugs to treat candidiasis (24).

To reduce the incidence and mortality of opportunistic fungal infections, experts agree that we need (i) improved diagnostics to allow more rapid implementation of appropriate therapies, (ii) more effective antifungal agents associated with less severe side effects, and (iii) to develop immunotherapies based on our mounting knowledge of antifungal immunity (10, 25). These same strategies could be used also to reduce the recurrence and severity of RVVC episodes. We hereby argue that an anti-*Candida* vaccine, as long as it was effective in the populations of patients it is intended to protect, could help reduce the global burden of both systemic and vaginal candidiasis bringing about substantial socioeconomic benefits.

In this review, we first provide an up-to-date overview of various efforts that have been attempted so far in the quest of an anti-*Candida* vaccine, which has remained elusive. We next critically assess potential common problems that might have hindered such efforts and finally propose a few tentative solutions to overcoming those problems.

## Current Anti-*Candida* Vaccine Landscape

In the last decades, several different anti-*Candida* vaccines have been proposed but only few of them have been tested in clinical trials (Table 1). Here, we will summarize the current anti-*Candida* vaccine landscape and discuss advantages and disadvantages of each vaccination strategy.

**Table 1:** Summary of past and current anti-*Candida* vaccine candidates.

Vaccine category	Description	Clinical trial stage	Protection (*C. albicans* infection site)	Cross-protection (non-*C. albicans* organisms)	Reference
LA	Hyphal-defective *C. albicans* strain PCA-2		SYS	*Staphylococcus aureus*	(26)
LA	MAP kinase-defective *hog1*Δ *C. albicans* strain CNC13		SYS		(27)
LA	Cell wall-defective *ecm33*Δ *C. albicans* strain RML2U		SYS		(28)
LA	Filamentation-repressible *C. albicans* strain tet-NRG1		SYS		(29)
LA	Yeast-locked *C. albicans* strain *cph1*Δ/*efg1*Δ		SYS		(30)
LA	Heat-killed or live *Saccharomyces cerevisiae* clinical strains		SYS	*Aspergillus fumigatus, Cryptococcus grubii, and Coccidioides posadasii*	(31–34)
REC	Recombinant N-terminus of *C. albicans* Als1p		SYS, VAG, OR		(35, 36)
REC	Recombinant N-terminus of *C. albicans* Als3p		SYS, VAG	*S. aureus*	(37, 38)
REC	Recombinant N-terminus of *C. albicans* Als3p plus alum adjuvant (NDV-3)	Phase II	SYS, VAG	*S. aureus*	(39)
REC	Recombinant *C. albicans* Sap2p		VAG		(40)
REC	Recombinant *C. albicans* Sap2p (PEV-7)	Phase I	VAG		(41)
REC	Recombinant *C. albicans* Hsp90p (Mycograb)		SYS		(42)
REC	Recombinant *C. albicans* Hyr1p		SYS	*C. glabrata, C. krusei, C. parapsilosis and C. tropicalis*	(43)
EX	Crude cell wall extract		SYS		(44)
GC	Mannans and peptide conjugates		SYS		(45, 46)
GC	β-glucan conjugated with MF59 adjuvant		VAG		(47)
GC	Laminarin conjugated with diphtheria toxoid (Lam-CRM197)		SYS, VAG	*A. fumigatus*	(48)

*LA, live attenuated; REC, recombinant; EX, extract; GC, glycoconjugate; SYS, systemic; VAG, vaginal; OR, oral*.

Historically, vaccines are developed to boost antigen-specific immune responses and elicit protective immunological memory against specific pathogenic strains. In the case of *Candida* species, most of the efforts have been focused around the most common species, *C. albicans*. Different strategies have been used to immunize hosts against candidiasis: (i) live-attenuated strains, (ii) recombinant proteins, and (iii) glycoconjugates.

### Live-Attenuated Vaccines

The first ever reported vaccine, Edward Jenner’s famous vaccinia virus, was a live virus causing cowpox in cattle but protecting humans against smallpox (49). By all definitions, it was a live-attenuated vaccine, which caused only a very mild form of the disease in humans while at the same time raising long-lasting protective immune memory. Up to this date, most currently available antiviral vaccines (e.g., yellow fever, measles, varicella) and some antibacterial ones (e.g., BCG) are essentially live-attenuated versions of their pathogenic counterparts. Because of this long history of success, it is not surprising that several studies have ascribed various attenuated strains of *C. albicans* the ability to confer protection against candidiasis.

The morphogenetic transition between the conidial (yeast) and hyphal (filamentous) form is one of the most important virulence factors in *C. albicans* (50); for this reason, the absence of filamentation is a common characteristic of attenuated strains of this species. Several *C. albicans* mutants associated with no or low virulence, including PCA-2 (incapable of yeast-hyphae conversion), CNC13 (deleted in the MAP kinase *HOG1*), RML2U [deleted in the cell wall protein (CWP) gene *ECM33*], and tet-NRG1 (in which the filamentation repressor *NRG1* can be overexpressed by doxycycline), have been used to immunize mice and shown to protect them from a subsequent lethal systemic infection with a virulent strain (26–29). Also, the *C. albicans* double-mutant *cph1*/*efg1*, which is severely impaired in hyphal morphogenesis, partially protects mice from systemic infection by a wild-type *C. albicans* strain (30).

Not only *C. albicans* attenuated strains have been used to protect against systemic candidiasis, but also the generally regarded-as-safe baker’s yeast *Saccharomyces cerevisiae* has been successfully used to this end. Stevens and colleagues have described the use of heat-killed or live *S. cerevisiae* as a protective vaccine against *C. albicans* infection, but also against *A. fumigatus, Cryptococcus grubii*, and *Coccidioides* infection in a dose-dependent manner (31–34).

Despite these success stories at the basic research level, none of these vaccine candidates has progressed to clinical trials. Possible reasons include the fact that characterization of these strains is complex, that the stability of the virulence-attenuating mutations is not guaranteed, and that the use of live-attenuated vaccines is currently not recommended in immunocompromised patients due to their weaker immune defense and the higher probability to develop a disease.

### Recombinant Proteins

In contrast to live *Candida* vaccines (how attenuated they may be), recombinant vaccines are generally perceived as potentially safer to the human host, because they do not contain any infectious agent. This characteristic makes recombinant protein vaccines more suitable especially for the immunocompromised individuals that would most benefit from such a vaccine. In general, this strategy has focused on proteins expressed on the surface of the fungus, such as CWPs or adhesion proteins, to ensure that epitopes would be easily accessible and “visible” by the immune system. Efforts also often concentrated on proteins critical to virulence, such as hyphal-specific effectors, in order to mount immune responses specifically against the pathogenic forms of the fungus. Besides, current work has shown that the strongest adaptive immune memory responses are often directed toward hyphal-associated proteins.

#### Agglutinin-Like Sequence Proteins

Agglutinin-like sequence (Als) proteins are located at the surface of *C. albicans* and play important roles in the adhesion to human endothelial cells and in the development of invasive candidiasis (51). Because these proteins are both exposed on the fungal surface and important for invasion of host cells, several vaccines have been proposed against invasive candidiasis using recombinant versions of various Als proteins, including Als1p and Als3p, formulated with or without adjuvants.

The recombinant N-terminus of Als1 (rAls1p-N) was produced and purified from *S. cerevisiae* and used in combination with Complete Freund’s Adjuvant (CFA) subcutaneously and boosted with different doses of rAls1p-N with Incomplete Freund’s Adjuvant (IFA) at day 21 post-immunization; lethal challenge with live *C. albicans* showed a survival rate of 50–57% and a decrease in fungal burden (36). This vaccine was deemed effective and improved survival in both immunocompetent and neutropenic mice and in murine models of oropharyngeal candidiasis and *Candida* vaginitis (35).

Vaccination with the recombinant N-terminus of Als3 (rAls3p-N) induced a stronger antibody response and survival rate compared with the rAls1p-N vaccine, and was more effective than rAls1p-N in the murine model of oropharyngeal and vaginal candidiasis (37). Interestingly, it showed also protection against *S. aureus* infection (38), suggesting the existence of shared immune epitopes between these distantly related species; consistently, *Candida* Als3p is structurally similar to a clumping factor of *S. aureus* (52).

Bar et al. identified an antigenic peptide by immunoproteomic approaches called pAls, widely conserved in many non-*Candida* species; this epitope mediates T-cell-dependent protection from invasive candidiasis. This pAls epitope is found in the Als3 protein and the authors suggest an implication of this epitope in the protective effect of the Als3 vaccine (53).

NDV-3A, a rAls3p-N vaccine formulated with Alhydrogel adjuvant, has been tested in a phase-I clinical trial. The trial was performed on 40 healthy volunteers and showed an increase in antibody titers at two different doses, as well as increased cytokine responses and IgG and IgA_1_ titers in the revaccinated subjects (39). A few adverse events have been described, but overall the vaccine was well tolerated by the subjects and hence the results were deemed as promising. Like the version of rAls3p-N vaccine without adjuvant, NDV-3 also showed activity against *S. aureus* infection, further corroborating the idea that the vaccination with *Candida* antigens containing epitope homologs found in other organisms can be harnessed to generate “convergent immunity.” NDV-3A is now in a phase-II clinical trial to test the immunotherapeutic effect of the vaccine in women with RVVC.^1^

#### Secreted Aspartil Proteases

Secreted Aspartil Proteases (SAP) constitute a family of 10 proteins secreted by *C. albicans*, which are required for adhesion, epithelial, and endothelial invasion and fungal cell metabolism (54, 55). Sap2p is the most abundantly expressed SAP in *C. albicans* and its recombinant form has been used to immunize rats intravaginally or intranasally, either using cholera toxin as an adjuvant or without an adjuvant; the vaccination resulted in the clearance of the *Candida* vaginal infection (40). The same research group also developed PEV7, a truncated version of Sap2p (amino acids 77–400) incorporated into the lipid bilayer of influenza virosomes. Because preclinical data demonstrated that intramuscularly vaccinated rats showed protection against vaginal candidiasis (41), PEV7 has progressed to clinical trials as a therapeutic vaccine for the treatment of RVVC. In a randomized phase-I trial, the subjects, whether immunized *via* intramuscular injections or by intravaginal capsules, showed a strong B-cell-mediated immune response in vaginal and cervical samples. All volunteers showed a mucosal immune response with consistently high titers across the groups, the response was dose-dependent and no serious adverse events were reported.^2^

#### Heat Shock Protein 90 (Hsp90p)

Heat shock protein 90 is a highly conserved stress-induced chaperone, with key functions in setting cellular responses to stressful stimuli, which is indispensable for yeast viability and located in the cell wall of *C. albicans*. The 47-kDa carboxyl fragment of *C. albicans* Hsp90 had been identified as a *Candida* antigen in different studies (56). This antigen is very abundant and immunogenic, and the presence of antibodies against Hsp90 correlates with good prognosis, whereas low levels are associated with mortality (57). Various Hsp90 epitopes (LSREM, LKVIRK, and DEPAGE) have been identified using a phage-display library and shown to induce Hsp90-specific serum antibodies and to prolong survival in a mouse model of systemic candidiasis (42). A recombinant protein called Mycograb, consisting of cross-linked Hsp90 NKILKVIRKNIVKK peptide-binding variable domains of human antibody heavy and light chains, was constructed and expressed in *Escherichia coli*. When combined with amphotericin B, Mycograb produced significant improvement in patients with invasive candidiasis (58, 59). However, the drug was refused by the European Medicines Agency on the grounds of product safety and quality, and its ability to potentiate the effects of amphotericin B were later found to be non Hsp90-specific (60).

#### Hyphally Regulated Proteins

Hyphally regulated 1 (Hyr1) is a glycosyl phosphatidylinositol (GPI)-anchor mannoprotein that is expressed during hyphal formation on the cell wall of *C. albicans*. A recombinant version of the N-terminus of Hyr1 (rHyr1p-N) was produced in *E. coli* and used to immunize mice *via* subcutaneous injection with either CFA or aluminum hydroxide and boosted on day 21 with IFA. After 2 weeks, vaccinated mice were challenged with a lethal dose of *C. albicans* and non-albicans *Candida* (NAC) species. The vaccine was effective against infections by *C. albicans, C. glabrata, C. krusei, C. parapsilosis*, and *C. tropicalis* in both immunocompetent and neutropenic mice (43).

#### CWP Extracts

Not only purified recombinant proteins, but also crude *C. albicans* cell wall extracts, seem to be effective to protect against invasive candidiasis. In fact, subcutaneous immunization with β-mercaptoethanol-extracted *C. albicans* CWPs in combination with Ribi Adjuvant System (RAS) R-700 followed by a booster injected 21 days after the first immunization, conferred protection in 75% of immunized mice after a lethal challenge with *C. albicans* (44). However, such crude preparations are unlikely to progress to clinical trials due to the difficulty in characterizing and standardizing such complex vaccine formulations.

### Glycoconjugates

An alternative to protein-based vaccines is to use glycans commonly found in fungal cell walls but absent in host cells. In fact, the *Candida* cell wall represents a hub of pathogen-associated molecular pattern (PAMP)–pathogen recognition receptor (PRR) interactions that dictate downstream immune responses (61). For these reasons, various cell wall polysaccharides have been tested as vaccine targets against systemic candidiasis.

#### Mannans and Derivative Peptide Conjugates

Mannans are polymers of *O*-linked or *N*-linked mannosides attached to CWPs and constitute the outermost, and hence most accessible, layer of the *C. albicans* cell wall. While *O*-mannans are predominately recognized by TLR4 (62), *N*-mannans are recognized by a multitude of receptors, including the Mannose Receptor (MR), DC-SIGN, Dectin-2, Galectin-3, and Mincle (63). Classically, *Candida* mannans have been attributed immunesuppressive properties (64); but the discovery that *N*-linked mannans are critically required for recognition by DC-SIGN and MR on human dendritic cells (DCs) has established a rational for conjugating them to antigenic peptides. Consistent with this idea, mannosylated antigens were shown to be presented more effectively than non-mannosylated forms (65).

In one example, researchers have used computational epitope searches to select 6 peptides in *C. albicans* CWPs (fructose-bisphosphate aldolase; methyltetrahydropteroyltriglutamate; hyphal wall protein-1; enolase; glyceraldehyde-3-phosphate dehydrogenase; and phosphoglycerate kinase) and conjugated these peptides with β-mannans to generate the first series of fully synthetic glycopeptide vaccines against systemic candidiasis. Depending on the specific peptide, the immunized mice showed 80–100% survival and reduced kidney fungal burden after the challenge with *C. albicans* (45).

More recent studies showed than the vaccination with BSA-based conjugates bearing synthetic α-1,6-branched oligomannosides induced humoral responses in mice and induced production of potentially protective antibodies (46). Immunization with *Candida* mannan-derived branched 3,6-di-*O*-substituted α-oligomannosides conjugated to BSA (M5-BSA and M6-BSA conjugates) induced similar levels of CD19^+^ B lymphocytes, but the candidacidal activity of polymorphonuclear leukocytes induced by opsonization with M6-BSA antisera was higher than that with M5-BSA, thus revealing some important differences in their ability to induce an effective and protective immune response against *Candida* infection (66). A better mechanistic understanding of the type of immune responses elicited by various *Candida* mannans is therefore required before these compounds can progress to clinical studies.

#### β-Glucan and Derivative Conjugate Vaccines

Directly underneath mannans lies a hidden layer of β-glucans, which is an essential component of the *Candida* cell wall (67) and elicits Dectin-1-dependent innate immune responses that are critical for host protection against fungi (68). Moreover, β-glucans from the *C. albicans* cell wall are highly immunogenic, leading to epigenetic and metabolic reprogramming of monocytes/macrophages, elevated cytokine responses and to innate host protection against systemic candidiasis (69–71). This phenomenon was termed “trained immunity” (72) and is discussed further in Section “Trained Immunity—Toward the Next Generation of Vaccines?”

When used in combination with the MF59 adjuvant (oil-in-water emulsion of squalene oil), fungal-derived β-glucan showed protection against murine vaginal candidiasis but is currently not approved for use in humans (47). The conjugation of the β-glucan preparation Laminarin (a soluble Dectin-1 ligand derived from seaweed) with diphtheria toxoid (Lam-CRM197) resulted, in murine models, in significant protection against systemic candidiasis, as well as cross-protection against aspergillosis (48). In further studies, researchers conjugated the diphtheria toxoid with various other β-glucan preparations, such as curdlan (a high-molecular-weight β-1,3-linked polymer produced by bacteria) or synthetic oligosaccharides that contained either only linear β-1,3 linkages or also branched β-1,6 linkages, and combined it with the MF59 adjuvant. Interestingly, conjugates raising antibodies against β-1,3-glucans were protective in a mouse model of systemic candidiasis, while anti-β-1,6-antibodies appeared to reverse this effect (73). Overall these studies reveal the high immunogenicity of β-glucans but also a surprisingly complex relationship between antifungal immune responses and host protection against fungal infections. Moreover, different *Candida* species expose β-glucan on their surface to different degrees (74), questioning how broadly protective such vaccines would be against various candidiasis-causing agents. These complexities will need to be better understood, before these polysaccharides can be translated into safe and effective antifungal vaccines for human use.

## Common Challenges Faced so Far

The fact that *C. albicans* is thought to have coevolved with humans for at least the past 2,000 years (75), that it is a lifelong inhabitant of humans that colonizes the gastrointestinal (GI) tract since birth (76) and that is then transmitted between family members (77), poses a few conceptual and technical challenges in the development of an anti-*Candida* vaccine. On one hand, this long interaction history with the human GI tract suggests that these fungi might have evolved a series of mechanisms to escape various host immune defenses, including a high genetic, phenotypic and morphological plasticity (78). On the other hand, humans might have learned to recognize *Candida* species as commensals and might have therefore developed immune tolerance toward them—breaking this tolerance is expected to be both difficult and in some cases even counterproductive (79). Lastly, the highest risk group for *Candida*-related infections is represented by immunocompromised and immunosuppressed patients, i.e., a class of individuals that is intrinsically less responsive to immunization. Here, we will outline each of these issues in greater detail, and later we will attempt to offer some possible ways forward based on recent learning points.

### Zeroing in on a Moving Target

#### Morphological and Phenotypic Plasticity of *C. albicans*

**Figure 1:**
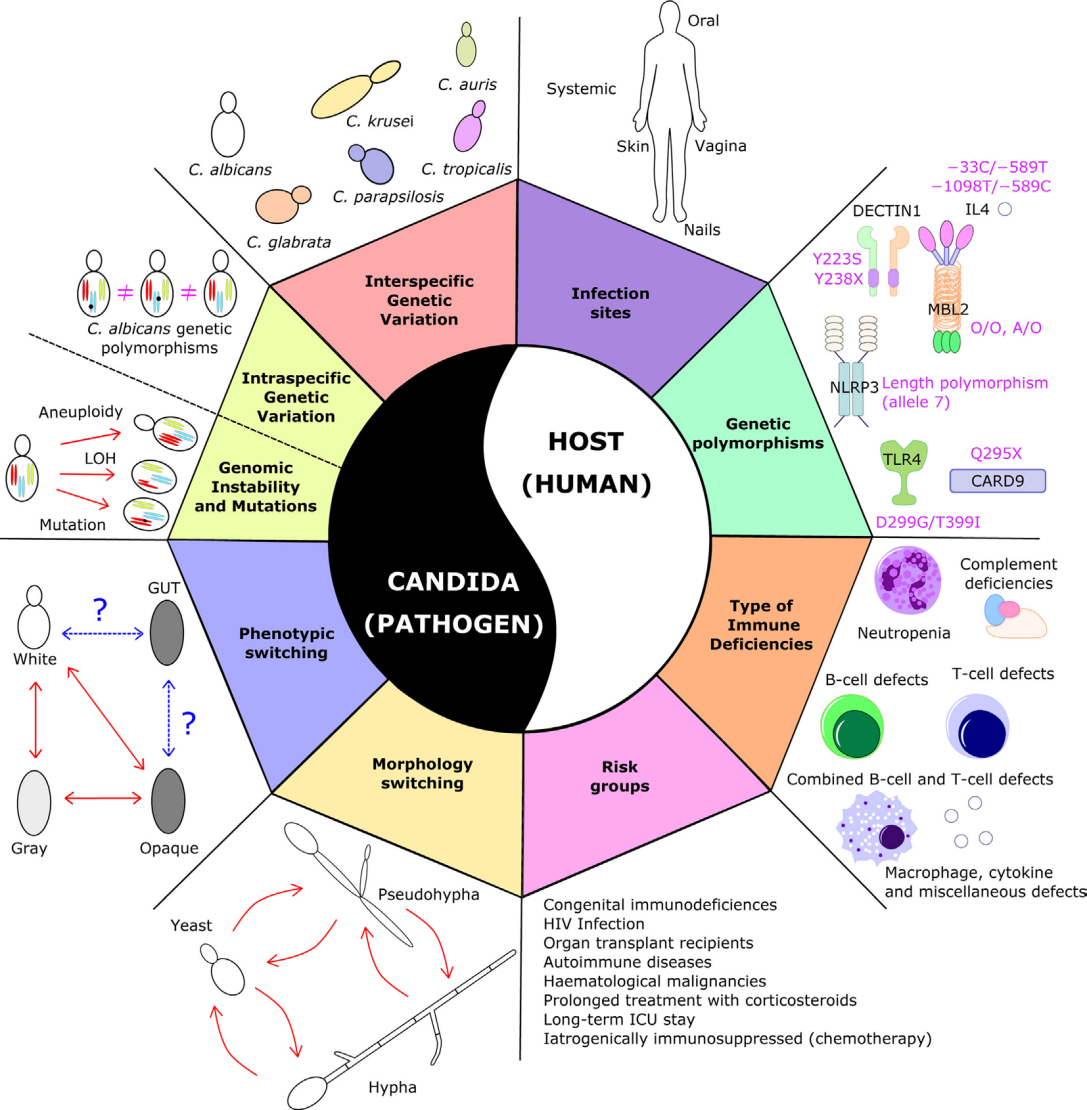
Sources of variability in *Candida*-related infections. The figure illustrates the plethora of variations at the level of the pathogen and the host, which pose serious challenges in the development of an antifungal vaccine. On the pathogen side, *Candida* species display large phenotypic, morphological, and genetic variation and dynamics; on the host side, candidiasis can occur in different types of patients, with different types of immune system dysfunction or carrying different genetic polymorphisms, and in different anatomical sites. Taken together, this broad and diverse clinical spectrum of the disease makes it difficult to design a “one-size-fits-all” vaccine that would protect all these different patients from all these different types of fungal infection.

A first difficulty encountered when designing an anti-*Candida* vaccine lies in the morphological and phenotypic plasticity of these fungi (Figure 1), which makes them almost “moving targets.” In fact, *C. albicans* is a polymorphic fungus that can reversibly transition between yeast, pseudohyphal and hyphal forms—a property that is closely linked to its evolutionary adaptation to life inside the human host. When *C. albicans* grows in its unicellular yeast form, it is commonly regarded as a harmless colonizer, while its switching to the hyphal form is related to pathogenesis, in that hyphae adhere to and invade epithelial cells resulting in extensive damage to host cells (80).

Interestingly, *C. albicans* isolated from the blood of patients with candidiasis often matches those recovered from the GI tract of the same patients (81), suggesting that *C. albicans* invades the bloodstream directly from the GI tract. How exactly the GI barrier is crossed is currently not completely understood, but it is thought to be mediated by or facilitated by epithelial tissue damage; and that *C. albicans* hyphal formation might be critical for this ability (82). Therefore, hyphal-specific or hyphal-associated CWPs such as Hyr1, Hwp2, Plb5, and Sod5 have all been proposed as vaccine target candidates (83).

However, vaccines against hyphal CWPs alone may not be sufficient to provide immune protection. While direct penetration into the mucosal surfaces by the hyphal form is important for invasion, widespread dissemination through the bloodstream and to the organs is thought to be facilitated by the yeast form (84). As both yeast and hyphae are detected in patients with candidiasis (85), and some mutants defective in filamentation displayed similar virulence as the wild type (86), it is the plasticity of this morphological switch, rather than the hyphal form alone, that appears to critically underlie the virulence of *C. albicans*. The variety of different forms of *C. albicans* that exist in various organs may represent a further challenge in vaccine development. For example, in mouse infection models, while *C. albicans* is mainly observed in the hyphal form in the kidney, it actually persists as a yeast in the spleen and the liver (87). To achieve sterile protection, the ideal vaccine should be able to stimulate clearance of the fungus not only in the former but also in the latter organs.

A second form of plasticity in *C. albicans* is a heritable white-to-opaque phenotypic switch. Smooth and white colonies are observed during the white phase, while elongated and rod-like cells giving rise to flattened gray colonies appear in the opaque phase (88). Opaque cells are less virulent than white cells in mouse bloodstream infection model (89, 90) and undergo filamentation in response to stimuli that are distinct form white cells (91). The switching between white and opaque phenotypes may facilitate *C. albicans* to evade from host immune response, as opaque cells are less susceptible to be phagocytosed by macrophages (92) and also capable to evade killing by neutrophils (93).

Another phenotypic switch, known as gastrointestinally induced transition (GUT) and found as a result of *WOR1* overexpression, was recently described to confer increased fitness of *C. albicans* in the GI tract of antibiotic-treated mice (94). While GUT cells share some resemblance with opaque cells, such as a reduced virulent in mouse bloodstream infections, they appear to be transcriptionally distinct from both white and opaque cells, therefore likely representing an independent epigenetic state. Whether GUT cells also arise in mucosal or systemic infections in humans and whether these cells express specific factors that are important for disease remains to be determined, but their discovery nonetheless sheds light on additional layers of complexity in the morphological and phenotypic plasticity of this fungal pathogen.

As a whole, morphological and phenotypic plasticity renders *C. albicans* a “skilled transformer” (78). Therefore, vaccines directed against single targets expressed only in a particular fungal form may not be sufficient to raise protective immune responses against the entire arsenal of antigens and virulence factors that are dynamically presented to the host in different organs at different times.

#### Genomic Plasticity of *Candida albicans*

*Candida albicans*, apart from morphological and phenotypic transitions, also displays a high degree of genomic plasticity including gross chromosome rearrangement, aneuploidy and loss of heterozygosity when exposed to different stresses. Such genomic changes equip *C. albicans* to rapidly adapt to an adverse environment by changing the copy number of specific genes on a given chromosome (95). One of the well-known examples is the identification of an isochromosome composed of two copies of the left arm of chromosome 5, which is associated with azole resistance (17) due to amplification of two resistance genes, *ERG11* and *TAC1* (96). Genomic variations are commonly observed in clinical *C. albicans* isolates, which includes copy number variations, chromosomal inversions, subtelomeric hypervariation, loss of heterozygosity and aneuploidy (97). Moreover, *C. albicans* with increased DNA content (aneuploidy) occurs at a higher frequency in clinical blood isolates when compared with mucosal isolates (98). This suggests that acquisition of aneuploidy, and the consequent copy number alteration of specific genes, may provide a rapid mechanism to modify the expression pattern of certain antigens that were originally targeted by a certain vaccine.

#### Non-albicans *Candida* Species

The genus *Candida* represents a highly heterogeneous group of >50 known species. Nevertheless, >90% of the invasive *Candida* infections are caused by *C. albicans, C. glabrata, C. parapsilosis, C. tropicalis* or *C. krusei* (99). While this figure has remained largely unchanged, the relative ranking between these species has seen significant shifts in different regions over the past decades (100). While *C. albicans* is still considered the most common species causing candidemia, increasing rates of NAC species in candidemia have been reported worldwide (18, 101, 102). Moreover, significant variations of *Candida* species are detected in different patient groups and geographical regions (103). For instance, *C. glabrata* has emerged as an important pathogen in northern Europe, the United States and Canada with a higher rate of incidence in adults than in children, and lower in neonates (104). *C. parapsilosis*, instead, is more prominent in southern Europe, Asia, and South America and is mostly associated with low-birth-weight neonates and transplant recipients (105). Finally, *C. tropicalis* constitutes 20–45% of *Candida* isolates in the Asia-Pacific region (18) and invasive candidiasis due to this species is commonly associated with patients with neutropenia and malignancy (106). Recently, the emergence of *C. auris*, first reported in 2009 in Japan, highlights a new challenge of antifungal treatment, as *C. auris* is often multi-drug resistant and also difficult to be diagnosed with standard laboratory methods, which is contributing to its rapid spreading over multiple countries (107). In addition to NAC, multispecies candidemia is also emerging as a threat (108). Taken together, these observations suggest that vaccines specifically against *C. albicans* alone may not be sufficient to provide protection against candidiasis caused by other emerging NAC species or mixed *Candida* species infections.

### Breaking Immune Tolerance Toward Fungi

A microbial ecosystem in the human intestine, known as the gut microbiome, harbors more than 100 trillion microorganisms and thus immunological tolerogenic responses are required in order to maintain gut homeostasis and prevent chronic inflammation (109). Immune tolerance toward human gut commensals, such as *Bacteroides fragilis* and certain *Clostridia* species, is maintained by regulatory T (Treg) cell responses (110–112). Apart from bacteria, *Candida* species are the most common fungal species found in the GI tract; it is therefore reasonable to assume that similar tolerance mechanisms might have evolved to regulate the commensal relationship between humans and fungi. One such mechanism, which likely resulted from the coevolution of bacterial microbiota, commensal fungi and host immune system, relies on the metabolic tryptophan-AhR pathway and 2,3-indoleamine dioxygenase (IDO) (113, 114). *C. albicans*, being a lifelong inhabitant of humans that colonizes the GI tract since birth (76), is able to induce IDO expression in DCs. IDO-expressing DCs then promote tolerogenic Treg responses, probably facilitating the switch from pathogenicity to commensalism in *C. albicans* (115).

The existence of immunological tolerance toward *C. albicans* and probably other *Candida* species poses two serious challenges for anti-*Candida* vaccine development. First, unlike obligate pathogens with no commensal relationship with humans, this tolerance represents an additional hurdle toward establishment of effective and protective immunological memory. Second, much of the clinical manifestations of *Candida*-related infections are more due to host-derived immunopathology than to pathogen-derived host damage (116, 117). For instance, mice lacking the chemokine receptor Ccr1, which is critical for neutrophil recruitment, display improved renal function during invasive candidiasis (118) and administration of Ccr1 antagonists reduces renal tissue injury and improves survival in mice challenged with systemic candidiasis (119).

It has thus been argued that a careful balance between immunity and tolerance must be established to maintain commensalism (79). Breaking host tolerance against fungi and the self-regulated equilibrium between Th17 and Treg responses might lead to undesired consequences, such as exacerbating fungal disease progression or other underlying inflammatory or autoimmune conditions.

### Vaccinating Patients With No Immunity

Fungal infections, especially invasive ones, most frequently occur in individuals with compromised or suppressed immunity. Hence, it appears that the patients that would mostly benefit from a future antifungal vaccine are also those least likely to respond to it. Is such an effort then even justified? Or is it doomed to fail from the start? Answers to these questions are more complex than one might expect.

Efficacy and safety are always a primary concern in any vaccine development effort, and especially so in vulnerable subjects such as patients with lowered immune defenses. As mentioned, the risk of *Candida* infection is especially high in patients with neutropenia, hematological malignancies, solid-organ transplants, prolonged treatment with corticosteroids, long-term ICU stay, chemotherapy and HIV infection (120). The ideal anti-*Candida* vaccine should possess all of the following properties: (i) zero risk of causing a *Candida*-related infection or to exacerbate the immunopathology associated with an ongoing or future fungal infection, (ii) be immunogenic enough to elicit protection in patients with little or no immunity, (iii) do not increase the risk of aggravating any underlying disease. While this may sound like a “catch-22,” vaccinating immunocompromised patients has been attempted with various degrees of success in the past for other kinds of infection.

For instance, a single-dose of the pneumococcal vaccine was recently deemed safe and immunogenic in children under active immunosuppressive therapy (121). However, low immunogenicity of the meningococcal vaccine was reported in solid-organ transplant recipients (122). Lower immunogenicity against the influenza vaccine is generally reported in immunocompromised patients when compared with healthy individuals (123). However, using a high-dose influenza vaccine was reported to be more immunogenic than the standard dose in children and young adults with leukemia or solid tumors, although not in those with HIV (124).

What is progressively becoming clearer is that a “one-size-fits-all” anti-*Candida* vaccine may never see the light. For example, a vaccine effective in preventing candidemia in neutropenic cancer patients might not be effective in T-cell-deficient HIV or transplant patients and *vice versa*. As a whole, one of the greatest challenges of any antifungal vaccine will be to deal with the large diversity of underlying disease states and of the associated types of immunosuppression that characterizes this highly heterogeneous risk group (Figure 1).

## Back to the Future: New Vaccine Strategies on the Horizon?

### Multivalent Vaccines

Some monovalent vaccines, i.e., those directed against a single strain or a single antigen, are very effective. A good example is represented by the measles vaccine, which has almost completely eradicated the disease in countries where it has been employed consistently throughout the population (125). However, the most recent vaccines tend to be multivalent, i.e., they carry multiple antigens of two or more strains/serotypes of the same pathogen. An example is the quadrivalent meningococcal vaccine that protects against 4 serogroups (A, C, W-135, and Y) of meningococci. The quadrivalent vaccine was shown to be more effective in reducing invasive meningococcal disease incidence when compared with the monovalent C vaccine (126). Another example is the 13-valent pneumococcal conjugate vaccine (PCV13) containing antigens from 13 serotypes of pneumococci. A replacement of the seven-valent pneumococcal conjugate vaccine (PCV7) with PCV13 covering additional six of the most prevalent serotypes that are not included in PCV7 successfully reduced the burden of pneumococcal disease in pediatric populations (127).

As *C. albicans* itself expresses a range of virulence factors and several NAC species also carry their own species-specific virulence factors, the recent development of univalent subunit vaccines may face practical obstacles. Considering above success stories of multivalent vaccines applied to bacterial infections, it has been argued that a better approach toward the development of an anti-*Candida* vaccine would be to simultaneously target multiple unrelated virulence-associated antigens (78). The predicted advantages include the lower probability of selecting “escape mutants” and a higher selectively against the pathogenic form of *C. albicans*, thus sparing the commensal—and perhaps beneficial—form of this gut inhabitant. In particular, it was proposed to combine a few of the univalent vaccines that have so far progressed furthest in clinical trials, such as the Als3 and the Sap2 vaccines (78).

More recently, a study reported that a newly identified cytolytic peptide toxin, Candidalysin, is secreted from *C. albicans* to induce epithelial cell damage and inflammation and to facilitate tissue invasion (128). Intriguingly, the mechanism of action of this novel virulence factor is partially uncoupled from the hyphal morphogenesis program, suggesting that a vaccine targeting both the former and the latter would be more effective than one targeting only one or the other. In general, as more knowledge is gained on the pathogenesis of *C. albicans*, further targets could be added to the list of antigens to be included in a multivalent formulation. At the same time, we can also predict that as the complexity of the vaccine increases, so will regulatory hurdles and manufacturing challenges. Nevertheless, testing the efficacy and safety of a combination of more and more antigens is likely going to be crucial for better vaccines against *Candida* infections.

### Trained Immunity—Toward the Next Generation of Vaccines?

**Figure 2:**
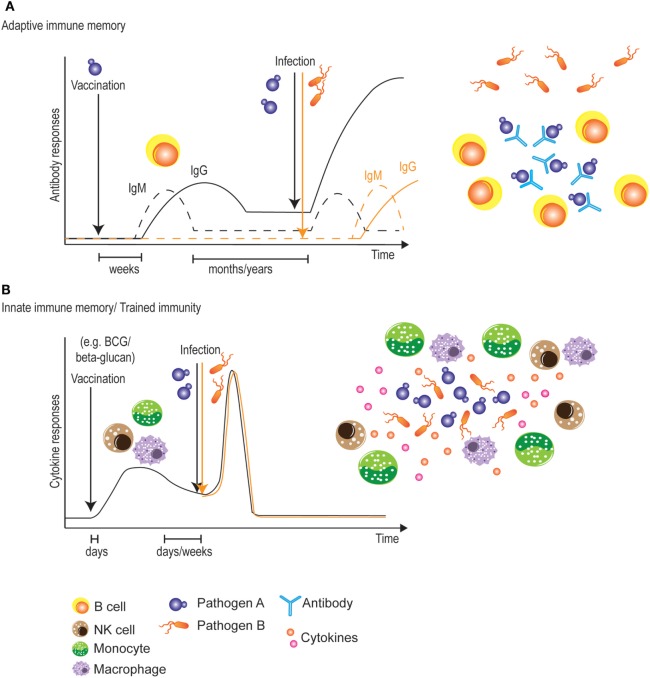
Vaccination strategies targeting adaptive or innate immune memory. **(A)** Classical vaccines initially induce a slow adaptive immune response. During this primary response, however, adaptive immune memory is raised, allowing for a more rapid and stronger secondary response upon encounter of the targeted pathogen. This protection is very long-lived, but is limited to the antigens present in the vaccine. **(B)** Most vaccines also induce a very rapid innate immune response. During this primary response, some vaccines also “train” innate immune cells, allowing for a stronger secondary innate response against pathogens. One advantage of this innate immune memory is the non-specificity of the secondary response, which could potentially lead to broadly cross-protective vaccines. A potential disadvantage would be the relatively shorter time window of protection.

The classic vaccination paradigm is based on adaptive immunity and the raising of long-lasting, protective B- and/or T-cell memory responses (129). As mentioned above, mounting protective antibodies or T-cell responses across different *Candida* strains and species may be challenging due to their large genetic, phenotypic and morphological variation and plasticity. In addition, the capacity to mount B- and T-memory responses may be impaired in immunocompromised or immunosuppressed patients such as HIV/AIDS or transplant recipients who are among the highest risk groups for candidiasis. Though innate immunity lacks most of the properties of classical immunological memory, various studies have demonstrated that certain vaccines induce a type of innate immune memory known as “trained immunity” (Figure 2), which is mediated by monocytes, macrophages or NK cells (72). For instance, BCG vaccination reduces mortality in low-birth-weight children (130, 131) and vaccination against measles reduces all-cause mortality in childhood (132), suggesting non-specific cross-protection against other infections (133).

Testing whether such innate memory responses could be stimulated also in adults, PBMCs obtained 2 weeks or 3 months after BCG vaccination were found to produce higher levels of pro-inflammatory cytokines such as TNF-α and IL-1β when restimulated *in vitro* with *C. albicans* (134). Such increased cytokine production capacity involved epigenetic changes mediated by increased H3K4 trimethylation in monocytes and was dependent on NOD2 and Rip2. Similarly, SCID mice vaccinated by BCG are protected against lethal *C. albicans* infection through a T and B lymphocyte-independent mechanism (134). Apart from the increased production of pro-inflammatory cytokines in monocytes, another study reported that BCG vaccination also enhances IL-1β production in human NK cells when stimulated with *C. albicans*. BCG-induced protection against disseminated *C. albicans* infection was shown to be partially dependent on NK cells in NOD-SCID-IL2Rγ^−/−^ mice (135). These studies warrant clinical trials to test if BCG vaccination could reduce the risk of candidiasis in high-risk patients.

Consistent with what reviewed in Section “Live-Attenuated Vaccines,” systemic infection of mice with an avirulent *C. albicans* strain was shown by Antonio Cassone’s group to confer protection against a subsequent challenge with a pathogenic *C. albicans* strain (26). The protection was shown to be non-specific, as cross-protection against *C. tropicalis* and *Staphylococcus aureus* was equally observed, and to be mediated by macrophage-like cells, as adoptive transfer of “plastic-adherent” cells was sufficient to confer protection against subsequent challenge with a virulent strain of *C. albicans*. Several years later, Mihai Netea’s group demonstrated that vaccination of both wild-type and RAG1^−/−^ mice, but not CCR2^−/−^ mice that lack circulating monocytes, with a sublethal dose of a virulent *C. albicans* strain similarly affords protection against reinfection with a lethal dose of the same strain, and dubbed the phenomenon “trained immunity” (69). Increased TNF-α and IL-6 levels from the trained monocytes were induced by β-glucan found in the *C. albicans* cell wall and sensed by Dectin-1 on host cells. In another study, mice trained with *Candida*-derived β-glucan showed increased serum levels of TNF-α and IL-6 when challenged by LPS four days later, but the response was transient and diminished after 20 days (136). To test the possibility of using β-glucan as a vaccine or “immune trainer” in humans, a pilot study was conducted by orally administrating β-glucan to healthy volunteers and subsequently testing innate immune responses in PBMCs restimulated *in vitro* with *C. albicans* (137). Enhanced innate immune responses as in the mouse model, however, were unfortunately not observed in this human study. This may probably be due to the solubility of β-glucan and the absorbability of β-glucan in the human GI tract. Further studies using different administration routes are still worth exploring, in order to establish whether β-glucan could one day be used as a vaccine to induce trained immunity against subsequent *Candida* infections (Figure 2).

## Concluding Remarks

With the increasing reports of multidrug resistance in several *Candida* species, prophylactic vaccination of at-risk patients likely represents a more effective long-term measure to reduce the growing incidence of *Candida*-related infections. The main challenges faced by *Candida* vaccine developers are the large variation and plasticity of these fungi, the existence of preestablished immunological tolerance and the difficulty in raising protective memory responses in patients with impaired adaptive immunity. In addition to multivalent vaccines, we propose that future vaccine development efforts should harness the growing mechanistic understanding of trained innate immunity, which might provide not only protection against candidiasis but also potentially cross-protection against a wide range of opportunistic infections. Further research on the mechanism, efficacy, and safety of raising trained immunity especially in immunocompromised patients would pave the way toward the development of a new generation of vaccines against *Candida*-related and other nosocomial infections.

## Author Contributions

GT and JR-C contributed equally as first authors. GT and JR-C conducted literature review and drafted the manuscript. NP conceptualized and oversaw the study and revised the manuscript. All authors read and approved the submitted version.

## Conflict of Interest Statement

The authors are inventors on a patent application related to the topic of this article.

## Footnotes

^1^https://www.clinicaltrials.gov/ct2/show/NCT01926028 (Accessed: April 17, 2018).

^2^https://www.clinicaltrials.gov/ct2/show/NCT01067131 (Accessed: April 17, 2018).
